# Automated Working Alliance Assessment in Psychological Counseling Using Gemini and XGBoost

**DOI:** 10.3390/e28060699

**Published:** 2026-06-17

**Authors:** Yuexi Li, Ningtao Sun, Zhuoxi Mai, Dalin Li, Guifang Fu, Xueling Yang

**Affiliations:** 1 College of Computer Science and Technology, Jilin University, Changchun 130012, China; yuexi23@mails.jlu.edu.cn; 2 School of Computer Science, Zhuhai College of Science and Technology, Zhuhai 519041, China; norton029@stu.zcst.edu.cn (N.S.); mzx@stu.zcst.edu.cn (Z.M.); 3 Department of Applied Psychology, Guangdong University of Foreign Studies, Guangzhou 510006, China; gffu@163.com; 4 Department of Psychology, School of Public Health, Southern Medical University, Guangzhou 510515, China; yhtyxl2006@126.com

**Keywords:** psychological counseling, working alliance, XGBoost, Gemini-2.5-Flash, hybrid feature representation

## Abstract

Session dialogue assessment based on machine learning is gradually becoming an effective solution for therapeutic alliance measurement which is an important factor for successful psychotherapy. However, most existing models assume clean and pre-structured dialogue transcripts, whereas real-world counseling documentation often contains heterogeneous case reports. This gap limits the applicability of current automated assessment models in realistic documentation scenarios. In this work, we propose a framework for automated working alliance assessment from complex, multilingual reports. First, language-specific BERT models are fine-tuned to process case reports across different languages, enabling accurate speaker role delineation and dialogue structuring. Second, Gemini-2.5-Flash is leveraged to annotate the dialogues with working alliance ratings. Third, a hybrid feature representation strategy is then developed to jointly capture linguistic style and semantic content from the counseling dialogues. Furthermore, an entropy-based mutual information analysis is conducted to identify the most informative linguistic features. Finally, the extracted hybrid features serve as inputs to XGBoost for alliance assessment. In experiments, the proposed framework shows better performance in the comparison with SOTA methods and generalization ability.

## 1. Introduction

The alliance between client and therapist is a crucial element in the process of psychological counseling. Previous studies have demonstrated that the quality of the therapeutic alliance significantly influences the outcomes of therapy [1]. The concept of the working alliance consists of three key components: the affective bond between the patient and therapist, their agreement on goals, and their agreement on tasks, as originally defined by Bordin [2]. Currently, the most widely used tool for assessing the therapeutic alliance is the Working Alliance Inventory (WAI) [3].

Driven by the growing demand for mental health services and the limitations of time-consuming manual review, research has increasingly turned to machine learning to automate therapeutic alliance assessment from the linguistic features of counseling dialogues. Goldberg et al. [4] utilized dialogue transcripts from therapy sessions, extracting features via TF-IDF [5] and Sent2vec, and applied linear regression (ridge regression) for alliance rating assessment. Zhou et al. [6] developed a model for predicting the therapeutic alliance following a client’s first session by incorporating various client and therapist attributes, such as gender, age, and therapeutic style. However, these approaches typically rely on pre-structured or clean dialogue transcripts. This assumption differs from many real-world counseling documentation scenarios. In routine psychological counseling practice, session information is often recorded as heterogeneous case reports rather than clean dialogue transcripts. A fairly substantial portion of reports combine therapist–client exchanges, narrative descriptions, assessment notes, intervention summaries, and treatment plans within the same document. In multilingual contexts, the problem becomes more challenging because documentation conventions, linguistic expressions, and speaker-role boundaries may vary across languages.

To separate dialogue and non-dialogue content and structure the reports for analysis, it is necessary to extract dialogue segments and accurately identify speaker roles. Building on this structured text, our study leverages natural language processing (NLP) to develop a framework for predicting the level of therapeutic alliance from such complex texts. The main contributions of our work are as follows:

(1) A generative data augmentation strategy is proposed, through which the PsyCase corpus is constructed with heterogeneous multilingual psychotherapy case reports to fine-tune BERTs. This approach successfully delineates speaker roles within complex, multilingual narrative texts across Chinese and English reports, establishing a structural foundation for automated alliance assessment.
(2) A computational framework for the automated assessment of therapeutic alliance from complex case reports is proposed and validated, demonstrating performance superior to existing benchmarks.

## 2. Working Alliance Assessment Framework Based on Gemini and XGBoost

**Figure 1:**
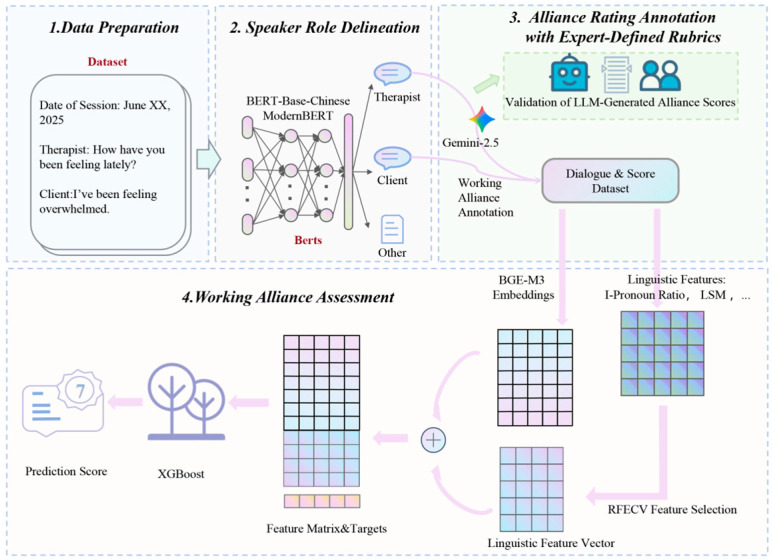
Overview of the automated working alliance assessment framework.

This research proposes a framework for the assessment of working alliance from psychotherapy case reports. The architecture of this framework is illustrated in Figure 1.

Specifically, the framework converts complex multilingual psychotherapy case reports into structured dialogue representations, which are used for downstream working alliance assessment. The framework consists of four stages: case report data preparation, speaker role delineation, rubric-guided alliance annotation, and hybrid linguistic–semantic score prediction.

### 2.1. Data Preparation

The framework begins with the construction of the PsyCase dataset. This dataset is generated by applying Gemini-2.5-Pro to transform raw dialogues from two existing open-source psychotherapy corpora, including the Chinese PsyDTCorpus [7] and the English FeedbackESConv [8], into fully formed case reports, as detailed in Section 3.1.1.

The purpose of constructing PsyCase is not to serve as the primary dataset for alliance score prediction, but to provide diverse case-report-style training data for speaker role delineation. To explain further, the final alliance prediction task is trained on Psy-Insight, whose dialogue content is sourced from human-written materials and then annotated using the rubric-guided procedure described in Section 2.3.

### 2.2. Speaker Role Delineation

A critical prerequisite for analyzing dialogue content within case reports is the accurate identification of speaker roles (“Client”, “Therapist”, “Other”). To perform this foundational task, pre-trained language models are fine-tuned for text classification. For multilingual processing, two widely used models are selected: ModernBERT-base for English and Bert-Base-Chinese for Chinese. The finetuning processes involve adding a simple classification head on top of the pre-trained BERT encoder. This head consists of a fully connected layer that maps the aggregated representation of the input text to an output dimension of three, corresponding to the target classes (“Client”, “Therapist”, “Other”). A softmax function is then applied to this output to generate a probability distribution for the final classification. During the finetuning process, the parameters of both the original BERT model and the new classification head are updated using a unified corpus integrating the PsyCase, Psy-Insight [9], and CPsyCounR [10] datasets (detailed in Section 3.1.1). This process trains the model to accurately delineate dialogue turns and their corresponding speakers from complex case report texts, thus converting raw text into structured data for downstream analysis.

### 2.3. Alliance Rating Annotation with Expert-Defined Rubrics

The core dataset for the final prediction task is Psy-Insight. This dataset is chosen over dialogue datasets automatically generated by large language models (LLMs), as its content, sourced from human-written books and blogs, offers a higher level of authenticity in its linguistic style and interactional dynamics. It initially lacks therapeutic alliance annotations. To generate rubric-guided supervisory labels for model training, an expert-guided annotation process is implemented. First, a detailed scoring rubric for the 11 subdimensions of the working alliance is developed in collaboration with Associate Professor Xueling Yang and Professor Guifang Fu, both experts in psychology.

These 11 subdimensions, listed in Table 1, are structured around Bordin’s three core components of Goals, Tasks, and Bond [2]. Specifically, dimensions “goal clarity,” “goal summary,” and “goal adjustment” map to the Goals component; “client participation,” “therapist support,” and “effective_communication” relate to the Tasks component; “positive emotions,” “therapist empathy,” “atmosphere label,” “self disclosure,” and “active listening” reflect the Bond component. This rubric provides clear definitions for the 11 subdimensions of the working alliance. For each subdimension, annotators are instructed to provide a score on a 1-to-7 rating scale, where 1 indicates strong disconfirming evidence and 7 indicates strong supporting evidence. Second, a two-tier strategy is also employed for large-scale annotation. Our primary annotator is the Gemini-2.5-Flash model, which is used to apply the expert-defined rubric to each session at a temperature of 0.1 to reduce output stochasticity. However, 65 sessions (approximately 6.8%) cannot be processed by Gemini-2.5-Flash due to its internal content safety filters. For these specific cases, DeepSeek-R1 [11] is utilized as a secondary, fallback annotator to ensure complete coverage of the dataset. Both models are prompted with the identical scoring rubric, few-shot scoring examples, and output format requirements to maintain consistency. The average of the 11 subdimensional scores is then used as the overall working alliance score, serving as the prediction target for our final model.

### 2.4. Working Alliance Assessment

In this section, the working alliance assessment is formulated as a session-level regression task. Each counseling session is represented by a hybrid linguistic–semantic feature vector and mapped to an overall alliance score.

#### 2.4.1. Hybrid Feature Engineering

The feature extraction operates on the structured client-therapist dialogue, which is processed by the speaker role delineation module. The core of our approach is a hybrid feature engineering strategy that combines two complementary sources of information: linguistic style and semantic content. The entire process is illustrated in the “Working Alliance Assessment” portion of Figure 1, which outlines two parallel streams for feature extraction.

To capture linguistic style, language-specific text processing is performed using the PKUSEG toolkit [12] for Chinese and the spaCy [13] library for English. Based on the resulting Part-of-Speech (POS) tags and tokens, we first compute utterance-level features inspired by Linguistic Style Matching (LSM) [14] using a hybrid approach: key function words such as pronouns and negations are extracted via predefined explicit word lists, while broader lexical categories including nouns and verbs are derived from the POS tags. These utterance-level features are summarized in Table 2A.

For example, the first-person singular pronoun usage ratio ($R_{\text{I-pronoun}}$), a key indicator of self-focus, is calculated for each utterance u. For an utterance containing a set of tokens $T_{u}$, this is formally defined as:

(1) $$R_{\text{I-pronoun}}\left(u\right)\;=\;\frac{\;\sum_{t\in T_{u}}I\left(t\;\in \;P_{I}\right)}{\left|T_{u}\right|},$$

where $P_{I}$ is the set of first-person singular pronouns (e.g., “I”, “me”, “my”), and $I\left(\cdot \right)$ is the indicator function. Similar ratios are computed for other linguistic categories ($R_{c}\left(u\right)$).

These utterance-level features are then aggregated to produce a representation for both the client ($R_{c,\mathrm{client}}$) and the therapist ($R_{c,\mathrm{therapist}}$). Specifically, as summarized in Table 2B, we derive three categories of session-level interaction features, including role-level statistics, talk-turn balance, and cross-role feature ratios. For the role-level statistics, the mean, sum, and standard deviation are simultaneously computed to capture baseline tendencies, total linguistic volume, and conversational variability.

To further model the interactional dynamics inspired by LSM principles, we calculate the Linguistic Style Dominance Ratio (LSDR) for each category c:

(2) $${\mathrm{LSDR}}_{c}\;=\;\frac{R_{c,\mathrm{client}}}{R_{c,\mathrm{therapist}}\;+\;\epsilon },$$

where ϵ is a small constant for numerical stability. The collection of all such individual and ratio-based linguistic features constitutes the final linguistic feature vector. To enhance robustness, Recursive Feature Elimination with Cross-Validation (RFECV) [15] is applied to the linguistic feature subset.

To quantify the informational contribution of each linguistic feature to alliance quality, we employ Mutual Information (MI) [16], a fundamental measure from information theory. For a linguistic feature X and the alliance score Y, MI is defined as:

(3) $$I\left(X;Y\right)=H\left(Y\right)-H\left(Y|X\right)$$

where $H\left(Y\right)$ denotes the marginal differential entropy of the alliance score and $H\left(Y|X\right)$ denotes the conditional entropy of Y given X, which quantifies the reduction in uncertainty about Y given knowledge of X. In practice, because the true underlying joint probability density functions $p\left(x,y\right)$ are unknown, we empirically estimate Mutual Information using a non-parametric k-nearest neighbor approach based on the KSG estimator [17]. A higher value of $I\left(X;Y\right)$ indicates that including feature X leads to a greater reduction in the uncertainty of Y, thereby providing a stronger predictive signal. Features are first ranked by their individual MI scores. RFECV then selects the optimal joint feature subset by maximizing cross-validated predictive performance. Table 3 summarizes the entropy reduction achieved by both strategies.

As shown in Table 3, the RFECV-selected subset achieves 47.1% (Chinese) and 40.7% (English) entropy reduction relative to the marginal entropy, consistently outperforming MI-ranked top-K selection. This confirms that the selected features capture substantial predictive information about alliance quality.

While linguistic features capture interaction style and the behavior of roles, the semantic embeddings provide complementary information about session content and contextual meaning. To capture semantic content, the multilingual BGE-M3 text model [18] is utilized. Each utterance is encoded into a 1024-dimensional vector, and these are aggregated via mean pooling to create a single, dense semantic representation for the entire session.

The linguistic and semantic feature vectors are then concatenated, merging two complementary feature sets into a unified and powerful representation. The resulting hybrid feature matrix, together with the corresponding alliance scores as targets, is used as the input to the XGBoost regression model.

#### 2.4.2. Predictive Modeling and Optimization

In this stage, the input to the predictor is a session-level fused vector, and the output is the predicted session-level alliance score. Moreover, our data are on a moderate scale, and the relationships among the features are nonlinear. As a result, this study adopts XGBoost as the predictor, because it is well-suited to heterogeneous tabular features.

The selected hybrid features are used to train an XGBoost regression model [19], an ensemble method based on decision trees. The model aims to minimize a regularized objective function, which balances the prediction error with model complexity. The regularized objective function L is defined as:

(4) $$L\left(\theta \right)\;=\;\sum_{i=1}^{n}{\left(y_{i}-\hat{y_{i}}\right)}^{2}+\;\sum_{k=1}^{K}\left(\gamma T_{k}\;+\;\frac{1}{2}\lambda \sum_{j=1}^{T_{k}}w_{\mathrm{kj}}^{2}\;+\;\alpha \sum_{j=1}^{T_{k}}\left|w_{\mathrm{kj}}\right|\right),$$

where the first term is the squared error loss over training samples, with $y_{i}$ being the true score and $\hat{y_{i}}$ the predicted score. The second term is the regularization penalty over all K trees in the ensemble. For each tree k, $T_{k}$ is the number of leaves, $w_{\mathrm{kj}}$ is the score of the j-th leaf, and γ, λ (L2 regularization), and α (L1 regularization) are complexity control parameters that are optimized using Optuna. Once the optimal parameter set is identified, the final XGBoost model is trained on the combined training and validation sets using this configuration.

## 3. Experiments

### 3.1. Identifying Dialogues and Speakers in a Case Report

#### 3.1.1. Datasets and Baselines

Building upon existing, open-source psychotherapy dialogue corpora, we construct the PsyCase dataset as part of our framework to support the development and evaluation of computational models for therapeutic alliance assessment. To generate the PsyCase dataset, we design a structured generation schema incorporating three pivotal dimensions: format, content, and style. This schema orchestrates the transformation process to ensure both diversity and consistency in the generated reports, operationalized through three distinct dimensions:

- Format. To ensure that the PsyCase dataset reflects the structural characteristics and informational richness of real-world psychotherapy records, we adopt three standardized documentation formats commonly used by clinical psychologists and counselors: SOAP (Subjective–Objective–Assessment–Plan) [20], BIRP (Behavior–Intervention–Response–Plan), and DAP (Data–Assessment–Plan) [21]. To enhance the structural diversity, two informal formats are also incorporated—narrative and timeline.
- Content. Generation prompts specify a broad range of clinical and psychological focuses, including intervention procedures, metaphor identification and interpretation, emotional tracking and annotation, cognitive-behavioral (CBT) analysis, psychodynamic analysis, person-centered process focus, behavioral pattern and function analysis, and cultural context analysis [22].
- Style. Four distinct writing styles are defined: academic, instructional, client-oriented, and reflective-practice. These three dimensions can be flexibly combined to generate a diverse set of case reports with coherent structure and professional expression.

Following the generation schema described above, the PsyCase dataset is constructed by applying the three-dimensional generation framework to two existing psychotherapy dialogue corpora: the Chinese PsyDTCorpus and the English FeedbackESConv dataset. Leveraging Gemini-2.5-Pro with prompts incorporating the defined format, content, and style dimensions, we generate 400 structured case reports from each corpus, resulting in a multiple-language dataset named PsyCase. The resulting PsyCase is subsequently integrated with the Psy-Insight dataset and the CPsyCounR dataset—both of which contain records combining dialogue and non-dialogue content. Detailed statistics of the datasets are presented in Table 4.

After dataset integration and deduplication, a unified and diverse corpus is formed and used to train the speaker role delineation model described in the following section. The model aims to distinguish therapist utterances, client utterances, and non-dialogue content within complex psychotherapy case reports. In this work, all datasets used are publicly available and consist of de-identified dialogue data or synthetically generated case reports. As no real personal identities or sensitive private information are involved at any stage of model training, the study complies with data privacy and research ethics requirements.

To comprehensively evaluate performance, several baseline models are employed for comparison, including XGBoost, Fully Connected Neural Network (FCNN), and Logistic Regression. All models are optimized using the Optuna framework [23] for automated hyperparameter tuning, ensuring a fair and consistent comparison. Baselines are trained on a feature set combining statistical properties, POS frequencies, and semantic embeddings.

#### 3.1.2. Experimental Settings and Evaluation Metrics

All experiments are conducted on a single NVIDIA GeForce RTX 3090 GPU (NVIDIA Corporation, Santa Clara, CA, USA). The dataset is partitioned into training, validation, and test sets using a 70:15:15 split ratio. Stratified sampling is employed to ensure that the class distribution remains consistent across all subsets. The model performance is evaluated using four metrics: Accuracy, Macro Precision, Macro F1 Score, and Macro AUC (One-vs-Rest).

#### 3.1.3. Experimental Results and Comparison

First, we evaluate the performance of the fine-tuned BERTs on the test sets. On the Chinese dataset, the BERT-Base-Chinese model achieves an accuracy of 98% and a macro F1 score of 97%. On the English dataset, the ModernBERT-base model achieves an accuracy of 97% and a macro F1 score of 94%. These results indicate that the fine-tuned BERTs effectively capture speaker-related information from complex case report texts across both languages.

To further analyze model performance, we compare the BERTs with several baseline approaches, including XGBoost, FCNN, and Logistic Regression. As shown in Table 5, the BERT-based models consistently outperform all baseline methods across both languages and all evaluation metrics.

### 3.2. Working Alliance Assessment

#### 3.2.1. Dataset

This study utilizes the Psy-Insight psychotherapy dialogue dataset. Detailed dataset information is provided in Table 6. The input features for model training consist of two main categories: linguistic features and embedding features. For POS tagging, language-specific tools are utilized to ensure high-quality annotations: the EN_CORE_WEB_TRF model from the spaCy framework is used for English, while the PKUSEG toolkit (version 0.0.25) is employed for Chinese. These tools enable accurate syntactic analysis of the dialogue texts, providing structured linguistic information for downstream feature extraction and modeling. Building on this foundation, a unified set of features—such as pronoun usage, non-fluency markers, and LSM categories—is extracted and aggregated at the session level for both the client and the therapist. The multilingual BGE-M3 model is used for semantic feature extraction across both languages.

In addition to Psy-Insight, we use PsyDial as an external dataset for generalization evaluation. PsyDial is a Chinese long-term counseling dialogue dataset derived from a real psychological counseling platform and reviewed under ethical and expert supervision. In this study, 300 sessions are randomly sampled from PsyDial for external evaluation. The PsyDial sessions have an average of 37.8 dialogue turns, and the average utterance lengths of clients and counselors are 33.7 and 31.1 Chinese characters, respectively.

#### 3.2.2. Experimental Settings and Evaluation Metrics

The dataset is partitioned into training, validation, and test sets using an 80:10:10 split ratio. To optimize the prediction model, we employ the Optuna framework for hyperparameter tuning. The search space is defined as follows: n_estimators $\in \left[200,\;2000\right]$ (step = 100); learning_rate $\in \left[10^{-3},\;0.3\right]$ (log scale); max_depth $\in \left[3,\;10\right]$; subsample $\in \left[0.6,\;1.0\right]$; colsample_bytree $\;\in \left[0.6,\;1.0\right]$; and L1/L2 regularization terms (alpha, lambda) and gamma all sampled uniformly on a log scale from $\left[10^{-8},\;1.0\right]$. Based on this search, the selected optimal hyperparameters for the Chinese subset are: n_estimators = 1000, learning_rate = 0.029, max_depth = 4, subsample = 0.88, colsample_bytree = 0.89, gamma = $2.8\times 10^{-7}$, lambda = $6.0\times 10^{-7}$, and alpha = 0.37. For the English subset, the selected parameters are: n_estimators = 1200, learning_rate = 0.014, max_depth = 3, subsample = 0.61, colsample_bytree = 0.62, gamma = 0.001, lambda = 0.39, and alpha = $2.5\times 10^{-8}$.

Model performance is evaluated using several regression-based metrics, including Mean Absolute Error (MAE), Root Mean Square Error (RMSE), statistical significance (*p*-value), Pearson’s Correlation Coefficient (r), and the Intraclass Correlation Coefficient (ICC).

#### 3.2.3. Performance Comparison

To comprehensively evaluate the effectiveness of different approaches for working alliance rating assessment, five representative models are implemented for comparison. The final evaluation results of these models on the test set are summarized in Table 7. Three of the models—XGBoost, ElasticNet, and BERT-RNN—utilize a combination of linguistic features and semantic embedding features as inputs, but differ in their core prediction algorithms, representing a gradient-boosted tree, a regularized linear regression model, and a deep sequence learning model, respectively. Specifically, the BERT-RNN model follows a hierarchical encoding architecture in which each utterance is encoded by a pretrained BERT model, the resulting CLS token representations are passed through a bidirectional LSTM with a hidden size of 128 per direction, and a soft attention mechanism aggregates the LSTM outputs into a session-level context vector that is concatenated with linguistic features and decoded by a two-layer MLP. For a broader comparison, we also replicate two methods from the literature: a Ridge Regression model with TF-IDF/Sent2vec features [4] and a “personae”-based SVR model [24]. Furthermore, we include GPT-4o-mini [25] as a zero-shot baseline to evaluate the capability of general-purpose large language models in directly annotating alliance ratings without task-specific supervised training.

These results demonstrate that the proposed model outperforms existing methods for predicting therapeutic alliance from dialogue content. For example, the replicated method of Goldberg et al. [4] achieves an MAE of 0.64 using TF-IDF features extracted from therapist utterances. Furthermore, although direct comparisons across different datasets are inherently limited, the correlation coefficients obtained in our study compare favorably with those reported in prior research. For instance, Aafjes-Van Doorn et al. [26] reported an ICC of 0.66 and a Pearson correlation of 0.70.

As shown in Table 7, the XGBoost model achieves the best performance across all evaluation metrics, which indicates that gradient-boosted ensemble methods are highly effective in leveraging mixed features for predicting therapeutic alliance scores. To assess the reliability of these results, 95% bootstrap confidence intervals are computed, and pairwise Wilcoxon signed-rank tests are conducted with XGBoost as the reference. Across both languages, XGBoost achieves statistically significant MAE reductions relative to TF-IDF_Ridge and the GPT-4o-mini baseline. Furthermore, on the English dataset, it also significantly outperforms SVR and BERT-RNN. Notably, the zero-shot GPT-4o-mini performs substantially worse than all supervised models in terms of error rate. Its predictions exhibit markedly reduced variance compared to the ground truth distribution, highlighting that general-purpose LLMs exhibit limitations in accurately assessing working alliance in the absence of task-specific adaptation. Furthermore, the BERT-RNN model performs relatively poorly. This is attributable to its large parameter count relative to the limited available training data, making it highly susceptible to overfitting and instability in small-sample contexts.

The performance differences between XGBoost and ElasticNet merit particular attention. Both models are trained using the same RFECV-selected feature set. Despite XGBoost’s consistent numerical advantage in point estimates across both datasets, pairwise tests show no statistically significant difference between the two models on the Psy-Insight dataset. This outcome can be attributed to the limited test set sizes, which limits the statistical power available to detect small effect sizes. In such a small-sample scenario, ElasticNet, as a highly regularized linear model, can adequately capture the variance using the well-selected RFECV features, masking the theoretical advantages of non-linear models.

Nevertheless, this lack of significance can be attributed in large part to the limited sample size. As demonstrated later in our cross-dataset generalization analysis (Section 3.2.6), when evaluated on the much larger PsyDial dataset, XGBoost significantly outperforms ElasticNet. This evidence suggests that the underlying relationship between linguistic features and alliance ratings is highly non-linear, and the superior capacity of gradient-boosted trees becomes decisively evident once sufficient statistical power is available.

#### 3.2.4. Ablation Study

An ablation study is conducted to validate the contribution of each feature type within the hybrid feature representation. Model performance is compared across four feature configurations: semantic embeddings only (Semantic Only), full linguistic style features only (Ling Only Full), RFECV-selected linguistic style features only (Ling Only Selected), and the proposed model integrating RFECV-selected linguistic features with semantic embeddings (Hybrid). The experimental results are presented in Table 8.

The results indicate that the XGBoost model with hybrid features consistently outperforms all single-feature configurations across all evaluation metrics, confirming the complementary nature of the two feature types. Pairwise Wilcoxon signed-rank tests comparing the Hybrid model with each ablation variant reveal that on the English dataset, the Hybrid model significantly outperforms the Semantic-Only configuration. Other ablation comparisons do not reach statistical significance on either dataset. This is primarily due to the limited test-set size. Nonetheless, the consistent numerical advantage of the Hybrid configuration across both languages, together with the statistically significant result on the English dataset, supports the complementary contribution of linguistic and semantic features. In particular, semantic features prove more informative than linguistic features in the Chinese setting, while the reverse pattern is observed in the English setting, suggesting that the two feature types emphasize different aspects across languages. Furthermore, RFECV-based feature selection consistently improves performance compared with the full linguistic feature set, indicating that removing redundant features enhances predictive accuracy without compromising essential information. Overall, the integration of linguistic style and semantic information serves a crucial role in improving working alliance assessment.

#### 3.2.5. Linguistic Feature Analysis

To identify the linguistic features that are most informative of therapeutic alliance quality, we apply the mutual information (MI) analysis described in Section 2.4.1 to the Psy-Insight dataset. Table 9 and Table 10 present the top 10 MI-ranked linguistic features for the Chinese and English subsets, respectively.

Mutual information analysis reveals both consistent and distinct cross-linguistic patterns in how linguistic features reduce the entropy of therapeutic alliance ratings. Across both languages, pronoun usage and verb-related features yield the highest information gain, indicating that personal reference patterns and action-oriented language serve as cross-linguistically robust channels for transmitting alliance-relevant information. In the Chinese subset, client-generated features dominate the top rankings. Client pronoun usage ranks first, followed by verb usage and specific first-person pronouns, demonstrating that the client’s active linguistic engagement is the primary source of entropy reduction when evaluating alliance quality. Significantly, interjection usage and negation also emerge as top-ranking features. This suggests that affective expressiveness and markers of resistance encode substantial, non-redundant information regarding the alliance state in Chinese-language counseling. Conversely, in the English subset, the therapist’s pronoun usage ranks first by a notable margin. This indicates that therapist linguistic accommodation provides the most discriminative power for alliance quality, playing a more prominent role in minimizing outcome uncertainty compared to the Chinese context. Furthermore, verb features from both participants appear in the top four, while the variability in therapist adjectives and adverbs also proves highly informative. This indicates that the lexical entropy of the therapist’s expressive language shares significant mutual dependence with the overall alliance state in English-language sessions.

**Figure 2:**
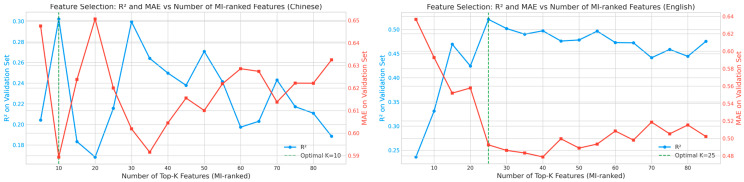
Top-K feature selection curves for the Chinese (**left**) and English (**right**) subsets, showing *R*^2^ and MAE on the validation set as a function of the number of MI-ranked linguistic features.

The Top-K cumulative performance analysis shown in Figure 2 provides insight into the minimal informative feature subset. In the English dataset, $R^{2}$ rises steeply from K = 5 to K = 25 and then plateaus, with the optimal subset at K = 25. In the Chinese dataset, the curve peaks early at K = 10 and exhibits greater fluctuation at larger K values. Despite the differing optimal K, both curves demonstrate that a small subset captures the majority of predictive information. Adding further features yields diminishing or even negative returns, confirming the presence of substantial redundancy in the full linguistic feature space.

**Figure 3:**
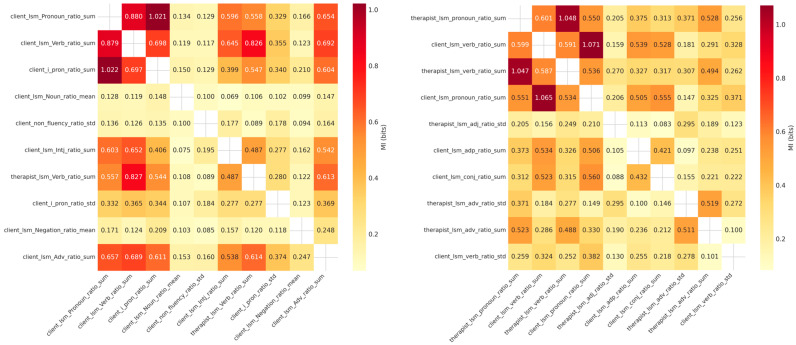
Pairwise mutual information heatmap of the top 10 MI-ranked linguistic features for the Chinese (**left**) and English (**right**) subsets, illustrating inter-feature redundancy within and across linguistic categories.

Furthermore, the pairwise MI redundancy heatmap in Figure 3 reveals two dominant sources of redundancy. The first is the subset–superset relationship. In the Chinese heatmap, client_LSM_Pronoun_ratio_sum and client_I_pron_ratio_sum share an MI of 1.02 bits because the first-person pronoun count is a strict subset of the overall pronoun count. The second is the cross-role co-variation in the same lexical category. Therapist_LSM_Verb_ratio_sum and client_LSM_Verb_ratio_sum exhibit MI values of 0.83 in Chinese and 0.59 in English, indicating that verb usage by one participant systematically predicts verb usage by the other, which quantitatively reflects linguistic style matching during therapy. In contrast, features capturing distributional variability such as client_non_fluency_ratio_std in Chinese and therapist_LSM_adj_ratio_std in English show consistently low MI with other features, suggesting that they provide unique and non-redundant information. These findings provide empirical justification for applying feature selection methods to reduce dimensionality without losing predictive power.

**Figure 4:**
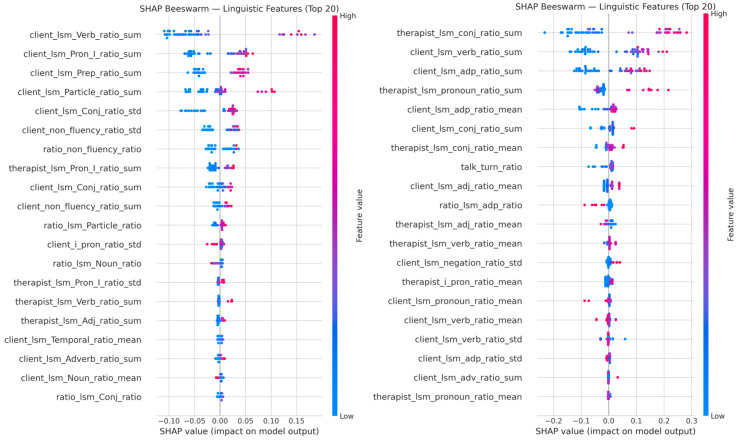
SHAP beeswarm plots of the top 20 linguistic features for the Chinese (**left**) and English (**right**) subsets on the Psy-Insight test set. Each dot represents one session; the x-axis shows the SHAP value (impact on model output); the color indicates the feature value.

To further validate the predictive contributions of linguistic features from a model-level perspective, we apply SHAP (SHapley Additive exPlanations) analysis to the trained XGBoost models on the Psy-Insight test set. Figure 4 presents the SHAP beeswarm plots for the Chinese and English subsets, showing the top 20 linguistic features ranked by mean absolute SHAP value.

In the Chinese subset, client_lsm_Verb_ratio_sum emerges as the most influential feature, with higher verb usage by the client associated with higher predicted alliance scores. client_lsm_Pron_I_ratio_sum and client_lsm_Prep_ratio_sum rank second and third, suggesting that client self-referential language and prepositional usage are strong positive predictors of alliance quality. Notably, client_non_fluency_ratio_std also ranks highly, indicating that variability in disfluency markers carries meaningful predictive signal, consistent with the MI analysis findings.

In the English subset, therapist_lsm_conj_ratio_sum is the dominant feature, with higher conjunction usage by the therapist strongly associated with higher alliance scores. This suggests that therapist discourse connectivity, defined as the degree to which the therapist linguistically links ideas within and across turns, is a key marker of alliance quality in English-language sessions. Client_lsm_verb_ratio_sum and client_lsm_adp_ratio_sum rank second and third, paralleling the client-side verb pattern observed in Chinese.

Overall, the SHAP analysis reveals both cross-linguistic consistencies and language-specific patterns in the predictive structure of the model, providing clinically interpretable evidence for how specific linguistic behaviors relate to therapeutic alliance quality.

#### 3.2.6. Generalization Capability Analysis

To further assess the robustness of our proposed framework, it is validated on a subset of 300 randomly sampled sessions drawn from the PsyDial dataset [27] without retraining. As described in Section 3.2.1, PsyDial differs from Psy-Insight in data source, language setting, and dialogue structure, and therefore provides a meaningful cross-dataset evaluation scenario. The reference alliance labels for the PsyDial subset are generated by Gemini-2.5-Flash using the same expert-defined rubric described in Section 2.3. The main domain differences between Psy-Insight and PsyDial are summarized in Table 11.

These differences indicate that the PsyDial evaluation involves cross-dataset transfer across different data sources, language settings, and dialogue structures. In this case, this experiment is intended to evaluate preliminary assessment of generalization under a consistent LLM-based annotation protocol, while acknowledging that further validation with independently human-rated labels is needed to establish clinical generalizability.

The results provide a preliminary assessment of cross-dataset generalization, as detailed in Table 12.

The XGBoost model achieved an MAE of 0.38 and an RMSE of 0.46 on the PsyDial subset. The predicted mean score (5.95) is close to the reference mean score (6.13), suggesting reasonable aggregate-level calibration. However, the statistical analysis shows a statistically significant but moderate correlations (Pearson r = 0.35, p < 0.05; Spearman ρ = 0.35, p < 0.05), revealing that the model captures part of the variance in the LLM-generated reference scores. These results provide preliminary evidence of cross-dataset generalization capability under the same annotation framework.

Comparative analysis further confirms the robustness of the XGBoost model. As shown in Table 12, the larger scale of the PsyDial dataset provides sufficient statistical power to firmly establish model differences. XGBoost significantly outperforms ElasticNet with a ΔMAE 95% CI of [0.329, 0.488] (p<0.001), confirming that its non-linear modeling capability is indeed superior to linear regularization when evaluated on a larger corpus. Table 12 also shows that when confronted with cross-dataset domain shifts, BERT-RNN fails to produce statistically significant correlations despite maintaining a comparable error rate. Linear models such as ElasticNet produce statistically significant correlations yet exhibit substantially higher error rates, indicating poor absolute calibration. Similarly, SVR and TF-IDF Ridge both achieve a significant Spearman correlation but not Pearson, with considerably higher error rates than XGBoost. These findings demonstrate that the integration of hybrid features with gradient-boosted ensemble learning yields a more stable framework under the current cross-dataset evaluation setting for psychotherapy dialogue assessment.

### 3.3. Validation of LLM-Generated Alliance Scores

To evaluate the validity of the alliance scores generated by LLMs, we conduct a validation analysis comparing the annotations produced by Gemini-2.5-Flash (with DeepSeek-reasoner as a fallback) with those provided by trained human raters on the entire Psy-Insight dataset. The human annotation process is supervised by Professor Guifang Fu, who has over twenty years of clinical and teaching experience in mental health and provides standardized training to all annotators. The annotators include undergraduate and graduate students majoring in psychology, all of whom receive formal instruction on applying the expert-defined scoring rubric across eleven alliance dimensions.

**Figure 5:**
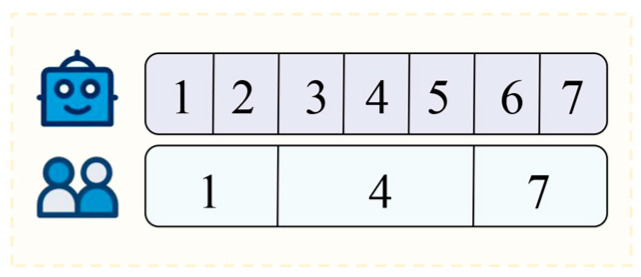
Illustration of the rating scale mapping process.

To ensure a fair comparison, both the human raters and the LLMs follow the same scoring rubric, as detailed in Table 1. However, the two groups differ in scoring granularity: the LLMs produce ratings on a 7-point scale (1–7), whereas human raters use a coarser 3-point scale (1, 4, 7). This is due to the challenge for human raters to maintain consistent and reliable distinctions across a 7-point spectrum for such a subjective task. To enable direct comparison, the LLM’s ratings are mapped to the same 3-point scale using threshold-based binning process, as illustrated in Figure 5.

We then analyze the agreement between the two sets of scores (LLM and human). To provide a comprehensive view, we examine both the overall score distributions and the level of direct alignment. The comparison includes descriptive statistics such as Mean, Standard Deviation (SD), Minimum, and Maximum. In addition, we calculate the MAE to quantify the average discrepancy and the Pearson Correlation Coefficient (r) to assess the strength of the linear relationship between the two rating sources.

As shown in Table 13, the validation results indicate a moderate correspondence between the LLM-generated annotations and those produced by trained human raters. The score distributions show that Gemini-2.5-Flash exhibits a mean score (M = 4.91) and standard deviation (SD = 1.03) that are broadly comparable to the human ratings (M = 5.37, SD = 0.95). The MAE of 0.80 and Pearson correlation coefficient of r = 0.59 (*p* < 0.001) indicate a statistically significant but moderate association between the two rating sources. It should be noted that these agreement metrics are computed after mapping LLM scores from the original 7-point scale to the coarser 3-level scale via threshold-based binning. This mismatch biases the reported r and MAE, as binning boundaries may produce apparent agreements or discrepancies that do not fully reflect the underlying evaluative relationship between the two rating sources. Although the use of a simplified 3-point scale for human raters results in some loss of granularity, this correlation indicates that the underlying evaluative trends are well preserved. Overall, these findings suggest that the LLM-generated scores can serve as useful rubric-guided supervisory signals for model development. However, they should not be regarded as fully equivalent to expert clinical ground-truth labels.

To rigorously evaluate the reliability of the human annotations, we randomly sampled a subset of 100 sessions and had them re-evaluated by other independently trained annotators. During the annotation process, human evaluators found that fine-grained clinical discrimination from raw text transcripts alone was highly challenging. Therefore, to ensure consistency, human annotators were instructed to rate sessions using three primary anchor points: 1 representing low, 4 representing moderate, and 7 representing high.

Based on this reliability subset, the inter-human Intraclass Correlation Coefficient is 0.52 (95% CI: [0.31, 0.67], *p* < 0.001), with a Pearson correlation of 0.58 and a mean absolute error of 1.00. This level of inter-rater agreement reflects the inherent complexity of assessing the working alliance solely from text transcripts. The discrete nature of the 1, 4, 7 anchor points inherently introduces large numerical penalties for single-category disagreements, thereby inflating the MAE and restricting the variance required for higher correlation coefficients.

In comparison, the agreement analysis between the Gemini-generated labels and the primary human annotators yielded r = 0.59 (*p* < 0.001) and an MAE of 0.80. We acknowledge that these values represent moderate agreement. However, viewed in the context of the inter-human baseline, this performance demonstrates that the LLM successfully achieves a level of consistency comparable to that of trained human evaluators. Consequently, these results validate that the structured, rubric-guided LLM annotation pipeline provides a reliable and highly scalable proxy for human clinical judgment, justifying its use for generating the prediction targets in this study.

## 4. Discussion

The experimental results demonstrate that the proposed framework achieves a strong and consistent performance on automated working alliance assessment, outperforming all baseline methods across both languages. A particularly informative comparison is that between XGBoost and ElasticNet, despite sharing identical input features, XGBoost substantially outperforms its linear counterpart, suggesting that the relationship between dialogue features and alliance ratings is inherently nonlinear and cannot be adequately captured by linear models. This advantage is further reinforced by the hybrid feature representation, which integrates linguistic style features with dense semantic embeddings to provide a richer characterization of the therapeutic interaction. Additionally, applying RFECV to the linguistic feature subset helps suppress noise and enhance model robustness by retaining only the most predictive features.

In addition to predictive performance, the validity of the LLM-based annotation pipeline is supported by the agreement analysis between Gemini-2.5-Flash and trained human raters (r = 0.59, *p* < 0.001, MAE = 0.80). Moreover, the LLM annotator follows a structured, expert-developed rubric grounded in Bordin’s three core components of the working alliance, reducing the risk of unconstrained or idiosyncratic model judgments. Overall, these results indicate that structured, LLM-assisted annotation offers a scalable and scalable rubric-guided annotation strategy for labeling psychotherapy process data.

Despite these promising results, several limitations warrant acknowledgment. First, an inter-human ICC of 0.52 reflects the inherent complexity of assessing the working alliance solely from text transcripts. This moderate inter-rater reliability highlights an intrinsic degree of noise in the ground-truth labels, which constrains the theoretical performance ceiling of any automated predictive model. Second, the scale of the psychotherapy datasets remains relatively limited, especially for training deep learning models and conducting multilingual evaluation. The Psy-Insight dataset contains 431 Chinese sessions and 520 English sessions, which may not fully cover the diversity of counseling styles, client concerns, and therapist intervention patterns. This limitation may partly explain the weaker performance of data-hungry models such as BERT-RNN, which typically require larger training corpora to generalize effectively. Although the proposed XGBoost model is more suitable for moderate-scale tabular features, the current dataset size still limits the strength of robust generalization claims. Furthermore, our framework relies partially on the synthetically structured PsyCase dataset, introducing a theoretical risk of learning LLM-generated artifacts. Future work should therefore prioritize expanding the real clinical corpus and examining the association between automated alliance scores and objective therapy outcomes to validate their clinical utility. Regarding practical deployment, prediction errors in regression-based alliance assessment carry asymmetric clinical risks: underestimating alliance quality may prompt unnecessary clinical intervention, whereas overestimation risks leaving genuinely at-risk cases undetected. Furthermore, careful consideration of data privacy protection remains essential, particularly given the sensitive nature of psychotherapy records.

## 5. Conclusions

This study proposes a framework for automated therapeutic alliance assessment from complex case reports. We demonstrate that by constructing the PsyCase dataset and leveraging LLMs for annotation, the proposed methods achieve strong performances. Our model, which processes case reports containing therapist–patient dialogue, can be integrated into clinical documentation workflows. After a session note is finalized, the system could automatically generate a consultation-alliance rating assessment, offering clinicians an additional interpretive indicator for identifying cases that may require closer attention to therapeutic engagement. In this role, the model serves as a supplementary analytic component that supports systematic reflection on interaction patterns within routine clinical practice. This research offers a novel approach for the intelligent evaluation of psychotherapy quality and contributes to the advancement of AI-assisted mental health interventions. While our findings are promising, certain limitations remain, pointing to avenues for future work. Significantly, the XGBoost model achieves robust performance, which underscores the effectiveness of our hybrid feature engineering strategy.

## Figures and Tables

**Table 1:** Description of the 11 subdimensions of working alliance.

No.	Subdimension	Description
1	Goal Clarity	The client clearly expressed the goals of the counseling.
2	Goal Summary	The therapist summarized and confirmed the client’s goals.
3	Goal Adjustment	Both parties discussed and adjusted the counseling goals together.
4	Client Participation	The client actively participated in the counseling process.
5	Therapist Support	The therapist provided effective guidance and support.
6	Effective Communication	Both parties were able to communicate effectively.
7	Positive Emotion	The client expressed positive emotions, such as hope, trust, and gratitude.
8	Therapist Empathy	The therapist expressed empathy and understanding.
9	Atmosphere Label	The conversation atmosphere was relaxed, natural, and sincere.
10	Self Disclosure	The client was willing to disclose themselves and share inner feelings.
11	Active Listening	The therapist demonstrated active listening and responsive engagement.

**Table 2:** (**A**) Definitions of utterance-level linguistic features; (**B**) Derived session-level interaction features.

**(A)**			
**Feature Category**	**Feature Name**	**Description**	**Purpose**
Pronoun Usage	i_pron_ratio	The proportion of first-person singular pronouns (e.g., ‘I’, ‘me’, ‘my’, ‘myself’) to the total word count per utterance.	To capture self-focused expression within each utterance.
	we_pron_ratio	The proportion of first-person plural pronouns (e.g., ‘we’, ‘us’, ‘our’, ‘ourselves’) to the total word count per utterance.	To capture collective or collaborative expression within each utterance.
Linguistic Style & Cognitive Process	non_fluency_ratio	The proportion of disfluency markers (e.g., ‘um’, ‘uh’, ‘er’, ‘hmm’) to the total word count per utterance.	To reflect hesitation, uncertainty, or disruptions in verbal fluency.
	lsm_{category}_ratio	The proportion of words belonging to specific Part-of-Speech (POS) categories (e.g., verbs, adjectives, adverbs, conjunctions) per utterance.	To characterize the linguistic style and lexical composition of each utterance.
(**B**)			
**Feature Name**	**Description**		**Purpose**
role-level statistics (_mean, _std, _sum)	For each utterance-level feature, the mean, standard deviation, and sum are calculated across all utterances for a specific role (client/therapist) within a session.		To capture role-specific linguistic tendencies, variability, and accumulated usage patterns within a session.
talk_turn_ratio	The ratio of the total number of turns taken by the client to the total number of turns taken by the therapist in a session.		To measure the balance of conversational participation between the client and the therapist.
cross-role ratios (ratio_{feature})	The ratio of the client’s mean value for a given linguistic feature to the therapist’s mean value for the same feature (client_mean/therapist_mean).		To quantify linguistic asymmetry between the client and the therapist for the same feature.

**Table 3:** Entropy reduction analysis on the Psy-Insight dataset.

Dataset (Language)	K	$H\left(Y\right)$	$H\left(Y|X_{\mathrm{MI}}\right)$	$\Delta H_{\mathrm{MI}}$	$H\left(Y|X_{\mathrm{RFECV}}\right)$	$\Delta H_{\mathrm{RFECV}}$
PsyCase (cn)	73	1.292	0.733	43.3%	0.684	47.1%
Psy-Insight (cn)	38	1.279	0.780	39.0%	0.758	40.7%

Note: $H\left(Y\right)$ is the marginal differential entropy (in nats). K is the number of features selected by RFECV. $\Delta H=H\left(Y\right)-H\left(Y|X\right)$ represents the entropy reduction.

**Table 4:** Statistical summary of the datasets.

Dataset (Language)	Case-Reports	Counselor-Utterances	Client-Utterances	Other-Utterances
PsyCase (cn)	400	7279	7279	17,013
Psy-Insight (cn)	431	2911	2865	1292
CPsyCounR(cn)	3127	2853	2901	32,907
Unified Corpus (cn)	3958	13,043	13,045	51,212
PsyCase (en)	400	1902	1731	19,115
Psy-Insight (en)	520	3097	3111	3090
Unified Corpus (en)	920	4999	4842	22,205

**Table 5:** Performance comparison of different models for speaker role delineation on Chinese and English datasets.

Model	Precision Macro	F1 Macro	Accuracy
XGBoost (cn)	0.94	0.93	0.95
BERT (cn)	0.97	0.97	0.98
FCNN (cn)	0.94	0.94	0.96
Logistic Regression (cn)	0.96	0.95	0.96
XGBoost (en)	0.91	0.91	0.95
BERT (en)	0.94	0.94	0.97
FCNN (en)	0.92	0.92	0.96
Logistic Regression (en)	0.91	0.91	0.95

**Table 6:** Statistics of Chinese and English counseling in the Psy-Insight dataset.

Category	Total	Therapist	Client
Cases (cn)	75	-	-
Sessions (cn)	431	-	-
Turn (cn)	5776	2911	2865
Avg.Utts./Case (cn)	77.01	38.81	38.2
Avg.Utts./Session (cn)	13.4	6.75	6.65
Cases (en)	114	-	-
Sessions (en)	520	-	-
Turn (en)	6208	3097	3111
Avg.Utts./Case (en)	54.46	27.15	27.31
Avg.Utts./Session (en)	11.94	5.95	5.99

**Table 7:** Summary of model evaluation metrics.

Model	MAE [95% CI]	RMSE [95% CI]	Pearson’s *r* (*p* < 0.05)	ICC
XGBoost (cn)	0.51 [0.42, 0.61]	0.61 [0.48, 0.74]	0.71	0.68
BERT-RNN (cn)	0.59 [0.44, 0.75]	0.79 [0.59, 0.99]	0.50	0.47
ElasticNet (cn)	0.54 [0.42, 0.68]	0.71 [0.53, 0.87]	0.58	0.57
TF-IDF_Ridge Regression (cn) †	0.67 [0.53, 0.81]	0.83 [0.64, 1.02]	0.32	0.25
SVR (cn)	0.63 [0.49, 0.77]	0.80 [0.61, 0.97]	0.38	0.26
GPT-4o-mini (cn) †	1.14 [0.94, 1.37]	1.35 [1.12, 1.56]	0.45	0.25
XGBoost (en)	0.45 [0.33, 0.58]	0.67 [0.44, 0.86]	0.76	0.71
BERT-RNN (en) †	0.67 [0.51, 0.83]	0.89 [0.69, 1.07]	0.55	0.52
ElasticNet (en)	0.53 [0.39, 0.69]	0.77 [0.52, 1.00]	0.67	0.66
TF-IDF_Ridge Regression (en) †	0.64 [0.49, 0.82]	0.90 [0.62, 1.16]	0.54	0.46
SVR (en) †	0.72 [0.55, 0.89]	0.95 [0.68, 1.20]	0.38	0.18
GPT-4o-mini (en) †	0.96 [0.80, 1.12]	1.13 [0.95, 1.28]	0.74	0.57

Note: Values in brackets represent 95% bootstrap confidence intervals based on 1000 resamples. † indicates models where XGBoost achieves a statistically significant MAE improvement based on paired two-sided Wilcoxon signed-rank tests and bootstrap CIs (i.e., lower bound of ΔMAE > 0).

**Table 8:** Ablation study results.

Feature	MAE [95% CI]	RMSE [95% CI]	Pearson’s *r* (*p* < 0.05)	ICC
Semantic Only (cn)	0.52 [0.40, 0.65]	0.67 [0.52, 0.82]	0.65	0.56
Ling Only Full (cn)	0.54 [0.41, 0.69]	0.70 [0.49, 0.90]	0.63	0.60
Ling Only Selected (cn)	0.52 [0.38, 0.66]	0.68 [0.48, 0.88]	0.63	0.57
Hybrid (cn)	0.51 [0.41, 0.62]	0.61 [0.48, 0.74]	0.71	0.68
Semantic Only (en) †	0.52 [0.40, 0.66]	0.74 [0.53, 0.94]	0.70	0.61
Ling Only Full (en)	0.51 [0.39, 0.63]	0.69 [0.52, 0.85]	0.73	0.69
Ling Only Selected (en)	0.49 [0.38, 0.62]	0.68 [0.49, 0.84]	0.76	0.69
Hybrid (en)	0.45 [0.33, 0.58]	0.67 [0.44, 0.86]	0.76	0.71

Note: Values in brackets represent 95% bootstrap confidence intervals based on 1000 resamples. All Pearson’s ***r*** values are statistically significant (*p* < 0.05). † indicates feature configurations that are significantly outperformed by the Hybrid model based on paired two-sided Wilcoxon signed-rank tests and bootstrap CIs (i.e., lower bound of ΔMAE > 0).

**Table 9:** Top 10 linguistic features ranked by MI for Chinese.

Rank	Feature	MI
1	client_lsm_Pronoun_ratio_sum	0.221
2	client_lsm_Verb_ratio_sum	0.214
3	client_I_pron_ratio_sum	0.191
4	client_lsm_Noun_ratio_mean	0.178
5	client_non_fluency_ratio_std	0.177
6	client_lsm_Intj_ratio_sum	0.170
7	therapist_lsm_Verb_ratio_sum	0.165
8	client_I_pron_ratio_std	0.150
9	client_lsm_Negation_ratio_mean	0.138
10	client_lsm_Adv_ratio_sum	0.138

**Table 10:** Top 10 linguistic features ranked by MI for English.

Rank	Feature	MI
1	therapist_lsm_pronoun_ratio_sum	0.269
2	client_lsm_verb_ratio_sum	0.191
3	therapist_lsm_verb_ratio_sum	0.176
4	client_lsm_pronoun_ratio_sum	0.169
5	therapist_lsm_adj_ratio_std	0.167
6	client_lsm_adp_ratio_sum	0.165
7	client_lsm_conj_ratio_sum	0.160
8	therapist_lsm_adv_ratio_std	0.156
9	therapist_lsm_adv_ratio_sum	0.142
10	client_lsm_verb_ratio_std	0.140

**Table 11:** Domain differences between Psy-Insight and PsyDial.

Aspect	Psy-Insight	PsyDial
Data Source	Books and blogs	Real counseling platform
Language	Chinese and English	Chinese only
Scale	431 CN sessions; 520 EN sessions	300 sampled sessions
Dialogue structure	Shorter session dialogues	Long-term dialogues; 37.8 turns/session

**Table 12:** Generalization Performance on the PsyDial Dataset.

Model	MAE [95% CI]	RMSE [95% CI]	Pearson’s *r* (*p* < 0.05)	Spearman ρ (*p* < 0.05)
XGBoost	0.38 [0.35, 0.41]	0.46 [0.43, 0.50]	0.35	0.35
BERT-RNN	0.40 [0.36, 0.43]	0.51 [0.47, 0.56]	n.s.	n.s.
ElasticNet †	0.79 [0.71, 0.87]	1.06 [0.96, 1.17]	0.25	0.28
TF-IDF_Ridge Regression †	0.66 [0.61, 0.69]	0.75 [0.71, 0.78]	n.s.	n.s.
SVR †	0.85 [0.81, 0.89]	0.94 [0.90, 0.98]	n.s.	0.16
GPT-4o-mini †	0.44 [0.39, 0.49]	0.59 [0.54, 0.65]	n.s.	n.s.

Note: Values in brackets represent 95% bootstrap confidence intervals based on 1000 resamples. † indicates baseline models that are significantly outperformed by XGBoost based on paired two-sided Wilcoxon signed-rank tests and bootstrap CIs (i.e., lower bound of ΔMAE > 0). n.s.: not statistically significant.

**Table 13:** Comparison of rating statistics between trained human raters and Gemini-2.5-Flash.

Rater Group	Mean	SD	Min	Max	MAE	Pearson’s *r*
Trained Students	5.37	0.95	1.55	7.00	0.80	0.59
Gemini-2.5-Flash	4.91	1.03	1.27	7.00		

## Data Availability

The publicly available datasets analyzed in this study are cited in the references. The newly generated PsyCase dataset presented in this study is not publicly available due to ongoing further research but is available on request from the authors.

## Abbreviations

The following abbreviations are used in this manuscript:

WAI	Working Alliance Inventory
TF-IDF	Term Frequency-Inverse Document Frequency
NLP	Natural Language Processing
BERT	Bidirectional Encoder Representations from Transformers
LLMs	Large Language Models
SOAP	Subjective–Objective–Assessment–Plan
BIRP	Behavior–Intervention–Response–Plan
DAP	Data–Assessment–Plan
CBT	Cognitive-Behavioral Therapy
FCNN	Fully Connected Neural Network
POS	Part-of-Speech
LSM	Linguistic Style Matching
LSDR	Linguistic Style Dominance Ratio
RFECV	Recursive Feature Elimination with Cross-Validation
XGBoost	eXtreme Gradient Boosting
MAE	Mean Absolute Error
RMSE	Root Mean Square Error
ICC	Intraclass Correlation Coefficient
SVR	Support Vector Regression
SD	Standard Deviation
